# Vitamin D status and parathyroid hormone assessment in girls with central precocious puberty

**DOI:** 10.1007/s40618-022-01838-y

**Published:** 2022-06-24

**Authors:** T. Durá-Travé, F. Gallinas-Victoriano

**Affiliations:** 1 Department of Pediatrics, School of Medicine, University of Navarra, Avenue Irunlarrea, 4, 31008 Pamplona, Spain; 2 Department of Pediatrics, Navarra Hospital Complex, Pamplona, Spain; 3 Navarrabiomed (Biomedical Research Center), Pamplona, Spain

**Keywords:** Central precocious puberty, Girls, Parathyroid hormone, Vitamin D status, 25-Hydroxyvitamin D

## Abstract

**Purpose:**

The objective of this study was to analyze vitamin D status and PTH concentrations in 6- to 8-year-old girls with central precocious puberty.

**Methods:**

A cross-sectional clinical and blood testing study (calcium, phosphorus, 25(OH)D and PTH) was carried out in 78 girls with central precocious puberty (CPP group), aged 6.1–7.9 years. A control group was recruited (137 prepubertal girls, aged 6.1–8.2 years). The criteria of the US Endocrine Society were used for the definition of hypovitaminosis D.

**Results:**

There were no significant differences in vitamin D status between both groups. There were no significant differences in 25(OH)D concentrations between CPP (25.4 ± 8.6 ng/mL) and control groups (28.2 ± 7.4 ng/mL). In contrast, PHT concentrations in CPP group (44.8 ± 16.3 pg/mL) were higher (*p* < 0.05) with respect to control group (31.0 ± 11.9 ng/mL). In CPP group, there was a positive correlation (*p* < 0.05) between PTH concentrations and growth rate, bone age, and basal estradiol, basal FSH, basal LH and LH peak concentrations.

**Conclusion:**

Vitamin D status in 6- to 8-year-old girls with CPP is similar to that in prepubertal girls. PTH concentrations were significantly higher in girls with CPP, and this could be considered as a physiological characteristic of puberty and, in this case, of pubertal precocity.

## Introduction

Vitamin D is currently considered a pleiotropic hormone. The biological actions of vitamin D are leaded by a specific nuclear transcription factor located in the target cells, called vitamin D receptor (VDR). Vitamin D binds to nuclear VDR, which interacts with other transcription factors and modulates the transcriptional activity of vitamin D-sensitive genes that play an important role in bone and mineral metabolism, as well as in other biological functions (non-classical effects of vitamin D) [1, 2]. VDR is scattered throughout most body cell and organs, including the skeleton and parathyroid glands, the immunity system (B and T lymphocytes, macrophages and monocytes), the endocrine system (pancreas, pituitary, and adrenal cortex), and the reproductive system (testicular tissue, sperm, prostate, ovaries, uterus, endometrium) [3]. The presence of VDR in both the pituitary gland and female reproductive tissue suggests that vitamin D could be involved in female fertility mechanisms as well as in the activation of the hypothalamic-pituitary–gonadal axis.

On the other hand, an inverse correlation between vitamin D and parathyroid hormone (PTH) concentrations has been reported by different authors [4–7]. Relatively asynchronous variations in PTH take place simultaneously with seasonal variations in vitamin D, a fact that presumably would allow maintaining constant calcium concentrations throughout the year and within the precise limits necessary for the performance of its metabolic and neuroregulatory functions. [8]. However, it is noteworthy that several authors have highlighted how PTH concentrations increase significantly in the age group of 8–10 years in girls, while they increase significantly in the age groups of 10–12 years in boys. Although these studies do not provide data concerning pubertal stage in the individuals, they do suggest the hypothesis of a relationship between the PTH concentrations and the timing of puberty [9].

Observational studies have focused on a potential relationship among vitamin D deficiency and in vitro fertilization outcome, polycystic ovary syndrome and endometriosis [10, 11]. There are hardly any studies on the relationship between vitamin D status and the timing of puberty. Furthermore, although several authors have suggested a possible association between vitamin D deficiency and the risk of central precocious puberty or earlier menarche [12–14], other authors have not found any correlation between vitamin D status and central precocious puberty [15]. Curiously, there are no reports on the relationship between PTH concentrations and precocious puberty.

The objective of this study was to analyze the vitamin D status and PTH concentrations in 6- to 8-year-old girls with central precocious puberty. We compared the blood concentrations of 25-hydroxyvitamin D (25(OH)D) and PTH between girls with precocious puberty and prepubertal girls. We hypothesized that girls with precocious puberty might have elevated PTH concentrations.

## Materials and methods

### Participants

The present work is a cross-sectional study (convenience sample) conducted in a sample of 78 girls, aged 6.0–7.9 years, diagnosed with idiopathic central precocious puberty (CPP group) (peak LH response to GnRH test > 5 IU/L), who followed a clinical examination and blood testing in the Pediatric Endocrinology Unit of the Navarra Hospital Complex (Pamplona, Spain) in the period January 2018–December 2020. All girls presented with Tanner stage 2 in breast development (appearance of the breast bud) and BMI SDS ranging from − 1.0 (15th percentile) to + 1.0 (85th percentile) (all those girls with overweight or obesity were excluded). The standardized protocol applied for the GnRH test has been previously published [16].

In addition to that, these parameters (clinical examination and blood testing) were determined in a control group that consisted of 137 healthy prepubertal girls (with no signs of pubertal development: Tanner stage 1), aged 6.1–8.2 years, with BMI SDS ranging from − 1.0 (15th percentile) to + 1.0 (85th percentile). These participants came from external consultations of the different pediatric subspecialties.

All participants (CPP and control groups) included in the study were Caucasian individuals living in Navarra, Spain. Neither participant (sample patients and control group) had been previously diagnosed from any illness that could affect bone health or growth (chronic pathologies, nutritional or others endocrine problems), nor had received any medication (antiepileptic drugs or glucocorticoids), vitamin D or calcium supplements.

Adequate information of the proceedings and potential implications was handed to the parents and/or legal guardians, and the corresponding consent was required prior to the inclusion in this study in all cases. The study was presented and approved after the evaluation of the Ethics Committee for Human Investigation at our institution (in line with the ethical standards stated in the Declaration of Helsinki 1964 and later amendments).

### Clinical examination

The anthropometric measurements were taken in accordance to a protocol that was previously published [17]. The information gathered from the participants included age, weight and height, body mass index (BMI), and bone age. Weight and height from every participant had been previously recorded by the pediatrician at the Primary Health Care Center (periodical health checkup), and enabled the calculation of growth rate (cm/year) 6–12 months before the current clinical evaluation. Individuals stood in underwear and barefoot for weight and height measurements. An Año-Sayol scale was used for weight measurement (reading interval 0–120 kg and a precision of 100 g), and a Holtain wall stadiometer for height measurement (reading interval 60–210 cm and precision 0.1 cm). Subsequent calculations allowed the evaluation of BMI by means of the following formula: weight (kg)/height^2^ (m). Bone age (BA) was calculated using RUS-TW2 method [18].

The SDS values for the BMI were quantified by applying the program Aplicación Nutricional, from the Spanish Society of pediatric gastroenterology, hepatology and nutrition. The graphics from Ferrández et al. (Centro Andrea Prader, Zaragoza 2002) were employed as reference charts [19].

### Blood testing

Blood sample for biochemical determinations (calcium, phosphorus, 25(0H)D and PTH) was collected after overnight fasting in all participants (in the CPP group, at the time of diagnosis and before the onset of any treatment). Calcium and phosphorus plasma concentrations were measured by colorimetric methods using a COBAS 8000 analyzer (Roche Diagnostic, Mannheim, Germany). In addition, 25(OH)D concentrations were estimated by a high-specific chemiluminescence immunoassay (LIAISON Assay, Diasorin, Dietzenbach, Germany) with intra- and interassay coefficient of variation of 4.2–9.5% and 7.6–2.1%, respectively, and functional sensitivity of 4.0 ng/mL; and PTH concentrations were determined by a highly specific solid-phase, two-site chemiluminescent enzyme-labeled immunometric assay using an Immulite analyzer (DPC Biermann, Bad Nauheim, Germany) with intra- and interassay coefficient of variation of 3.8–6.9% and 3.1–7.2%, respectively, and functional sensitivity of 5.0 pg/mL.

The distribution of individuals based on vitamin D serum concentrations followed the criteria of the United States Endocrine Society [20, 21]. In this way, 25(OH)D serum concentrations lower than 20 ng/mL (< 50 nmol/L) corresponded to vitamin D deficiency, 25(OH)D concentrations between 20 and 29 ng/mL (50–75 nmol/L) to vitamin D insufficiency, and concentrations equal to or higher than 30 ng/mL (> 75 nmol/L) to vitamin D sufficiency.

As it was a convenience sample, the statistical power of the study was not calculated a priori. However, a posteriori it was possible to verify that, given the differences observed between the variables analyzed with statistical significance (e.g.: calcidiol and PTH) between both groups (CPP and control), and even though the number of patients analyzed was relatively small, the calculated statistical power ranged from 84 to 87%.

### Statistical analysis

The results are displayed as percentages (%) and means (M) with the corresponding standard deviation score (SDS). Chi-square test was applied to compare percentages between CPP and control groups. Student’s *t*-test was used to compare mean values in the variables recorded between CPP and control groups. ANOVA or Kruskal Wallis test (non-parametric test) were implemented to compare mean values in variables according to the season of the year or vitamin D status. Pearson's test was used to quantify the degree of linear association between quantitative variables in both groups. Statistical analyses were performed using the program Statistical Packages for the Social Sciences version 20.0 (Chicago, IL, USA). Statistical significance was accepted when *p* value was < 0.05.

## Results

In CPP group, 25(OH)D concentrations exceeded 30 ng/mL (Vitamin D sufficiency) in 27 patients (34.6%), varied from 20 to 29 ng/mL (Vitamin D insufficiency) in 39 (50%) and were lower than 20 ng/mL (Vitamin D deficiency) in 12 (15.4%). In control group, 58 participants (42.3%) exhibited vitamin D sufficiency, 63 (46%) were vitamin D insufficient and 16 (11.7%) vitamin D deficient. There were no significant differences in the prevalence of hypovitaminosis D between both groups (Chi-square test).

Table 1 displays and compares mean values for the clinical characteristics and biochemical determinations between CPP and control groups. The average values for growth rate and PTH were significantly higher (*p* < 0.05) in CPP group. There were no significant differences in age, BMI SDS, calcium, phosphate and 25(OH)D between both groups.

Table 2 displays and compares the average values for biochemical characteristics whose determinations were made in the different seasons of the year between CPP and the control groups. There were not any statistically significant differences in the mean values of calcium and phosphorous concentrations between CPP and control groups for each season. There were seasonal variations in 25(OH)D concentrations (*p* < 0.05) in both groups, and the highest 25(OH)D concentrations corresponded to summer (CPP group: 32.4 ± 8.4 ng/mL and control group: 36.3 ± 7.7 ng/mL). There were not any statistically significant differences in the mean values of 25(OH)D concentrations between CPP and control groups for each season. There were seasonal variations in PTH concentrations (*p* < 0.05) in both groups. The lowest PTH concentrations corresponded to summer (CPP group: 37.7 ± 14.9 pg/mL and control group: 23.6 ± 6.5 pg/mL) and reached a maximum value in autumn (CPP group: 50.2 ± 17.1 pg/mL and control group: 35.1 ± 12.9 pg/mL). In the CPP group, mean values for PTH concentrations were significantly higher (*p* < 0.05) in every season of the year with respect to the control group.

Table 3 shows and compares the mean values for biochemical determinations in accordance to vitamin D status in PPC and control groups. No significant differences were found in calcium and phosphorus concentrations between different vitamin D status in both groups, and obviously 25(OH)D concentrations were significantly lower (*p* < 0.01) in deficiency and insufficiency than in vitamin D sufficiency in both groups. PTH concentrations were significantly higher (*p* < 0.01) in insufficiency and deficiency than in vitamin D sufficiency in both groups. In addition, there were no significant differences in calcium, phosphorus, and 25(OH)D concentrations in each vitamin D status between the two groups. However, PTH concentrations were significantly higher (*p* < 0.01) for each vitamin D status in the CPP group with respect to the control group.

Table 4 displays and compares the average values for clinical data and basal and stimulated serum concentrations of gonadotropins (LH and FSH) in accordance with vitamin D status in CPP group. There were not any statistically significant differences in age at onset, BMI SDS, growth rate, bone age, bone age advanced, basal estradiol, basal and stimulated LH and FSH between the different vitamin D status.

There was a negative correlation (*p* < 0.01) between PTH and 25(OH)D concentrations in both CPP (*r* = − 0.345) and control group (*r* = − 0.278). In addition, in CPP group, there was also a positive correlation (*p* < 0.05) between PTH concentrations and growth rate (*r* = 0.285), bone age (*r* = 0.539), basal estradiol (*r* = 0.288), basal FSH (*r* = 0.284), basal LH, (*r* = 0.412) and LH peak (*r* = 0.378). We did not find any significant correlation between 25(OH)D concentrations and pubertal features (BMI SDS, growth rate the year before puberty onset, BA, BA advance, basal estradiol, basal FSH, basal LH, FSH peak, and LH peak).

## Discussion

This study highlights that vitamin D status in 6- to 8-year-old girls with CPP is similar to that in control group (prepubertal girls), whereas PTH concentrations were significantly higher in girls with CPP compared to those in control individuals. Furthermore, our data revealed that girls with CPP also maintain seasonal variations in 25(OH)D and PTH concentrations, as it occurs in the control group. It should be noted that this is the first report that simultaneously analyzes vitamin D status and PTH concentrations in girls with CPP.

Vitamin D and PTH are well known because of their role in bone metabolism and calcium homeostasis. Their biological functions are widely complementary, contributing to the normal bone mass acquisition. The major physiologic function of vitamin D is to enhance the absorption of dietary calcium and phosphate, and to increase tubular reabsorption of calcium [1, 3]. On the other hand, under physiological conditions, PTH promotes bone mineralization through the effect on osteoblasts. During hypovitaminosis D or hypocalcemic phases, PTH concentrations increase and the osteoclast becomes its main target cell in bone; in this way, it activates bone resorption. The two processes do not occur independently, and the increase in bone resorption is accompanied by stimulation of osteoblast activity via a series of paracrine and autocrine mechanisms that result in an increase in bone turnover [22]. PTH concentrations are currently used as the best markers of bone turnover [9]. In accordance to several authors [4, 6, 7, 23, 24], we found a negative correlation between PTH and 25(OH)D concentrations, and this would be consistent with the physiological feedback mechanism of vitamin D on PTH secretion.

In contrast to other studies, we found no association between vitamin D status and CPP characteristics (clinical data, and basal and stimulated gonadopropins). To a large extent, this disagreement is possibly due to methodological reasons, since the sample in this study was entirely made up of Caucasian girls and, furthermore, all those overweight/obese girls have been excluded (cutaneous pigmentation and overweight/obesity have been associated with hypovitaminosis D) [25–28]. On the other hand, we cannot define a vitamin D status in a concrete population without referring to seasonal variations since, as it occurred in this study, the maximum concentration of 25(OH)D usually corresponds to the summer period, being significantly lower in the rest of the seasons of the year [4, 8, 29]. In this case, there was sufficient homogeneity in the seasonal distribution among the participants included in this study (CPP and control groups). However, previous studies that evaluated a possible association between vitamin D status and timing of puberty in girls have several limitations, since either they did not refer to race or season of the year in which the sample was collected [12–14], or did not include a group control [15]. In other words, this study allows us to analyze the results obtained avoiding confounding factors.

There is evidence that vitamin D has some effects on female fertility. VDR has been shown to be expressed in ovarian cells, indicating a role in steroidogenesis of sex hormones [10, 11]. In addition, VDR and 1α-hydroxylase are expressed in the endometrium, suggesting that it is an extra renal site of vitamin D synthesis and vitamin D action [30]. There are several studies that suggest that vitamin D status could contribute to hormonal dysregulation in patients with POCS; and it is even pointed out that vitamin D supplementation may be useful to improve fertility and metabolic alterations in women with POCS [31, 32]. Recent studies have reported that vitamin D is involved in ovarian steroidogenesis by modulating the expression of the 3β-hydroxy steroid dehydrogenase enzymes [33]; therefore, it could be suggested that a situation of hypovitaminosis D could rather condition a deficiency of gonadal steroids and, consequently, a delayed puberty. Experimental studies have revealed that peripubertal vitamin D sufficiency is important for an appropriate pubertal transition and maintenance of normal female reproductive physiology [34]. In this sense, it was expected that vitamin D status in girls with CPP were similar to that of the control group, as we found in this study. In addition, it should be remembered that we found no association between vitamin D status and CPP characteristics.

The main innovation of this study would be the fact that PTH concentrations in girls aged 6–8 years with CPP are increased in relation to the control group. And, curiously, it should be noted that our finding that PTH concentrations remained significantly increased throughout the year in the CPP group compared to the control group, even with seasonal variations. In addition, it can be said that PTH concentrations were also significantly higher—independent of vitamin D status—in girls with CPP with respect to the control group. Our data support the hypothesis that the high levels of PTH found in girls with CPP do not represent, as it is commonly assumed, an increase secondary to hypovitaminosis D.

During growth, the shape and structure of bones are continuously modified and renovated by modeling and remodeling processes. Maintaining normal bone turnover is important to achieve optimal peak bone mass and to optimize growth. This apposition-reabsorption process involves a positive balance during childhood and adolescence until the maximum peak of bone mass is acquired in the third decade of life, then moving to a neutral balance [35]. PTH concentrations are currently considered the best available marker of bone turnover. It is interesting to note that it has been reported that, in healthy children and adolescents, PTH concentrations increase significantly in girls in the age group of 8–10 years, whereas in boys it is significantly increased in the age groups of 10–12 years, leading to the hypothesis of a relationship between PTH concentrations and pubertal and bone growth spurt [9]. That is, the increase of PTH concentrations in these age groups, and with a clear sexual dimorphism, could be considered as a physiological characteristic of the pubertal period and, in this case, of pubertal precocity. There was a strong correlation between PTH concentrations and bone age in CPP group. Thus, these results support our working hypothesis that girls with precocious puberty might have elevated PTH concentrations as it appears to occur in the chronologically normal pubertal period. However, there is controversy regarding the relationship between vitamin D and bone turnover markers. In compliance with several studies [4, 6, 7, 23, 24], we found a negative correlation between the concentrations of PTH and 25(OH)D, and several authors have suggested that vitamin D deficiency could contribute through hyperparathyroidism secondary to accelerated bone turnover. However, we found that PTH concentrations were also significantly higher, regardless of vitamin D status, in girls with CPP compared to the control group; therefore, this hypothesis seems unreliable.

This study has some limitations. Unfortunately, bone alkaline phosphatase values as markers of bone turnover were not determined in the participants included in this study. It would have been very interesting to analyze its possible correlation with PTH concentrations, pubertal development and vitamin D status. Another limitation is that it would have been very suitable to have been able to compare the results obtained in this study with an additional group of girls with physiological pubertal development.

As a conclusion, vitamin D status in 6- to 8-year-old girls with CPP is similar to that in prepubertal girls, and we did not find any correlation between vitamin D status and CPP characteristics. Additionally, PTH concentrations were significantly higher in girls with CPP compared to those in control individuals, and could be considered as a physiological characteristic of the pubertal period and, in this case, of pubertal precocity.

**Table 1:** Clinical and biochemical characteristics in CPP and control groups (*M* ± SDS)

Items	CPP group (*n* = 78)	Control group (*n* = 137)	*p* value*
Age (years)	7.4 ± 0.5	7.5 ± 0.7	0.260
BMI SDS	0.33 ± 0.65	0.22 ± 0.75	0.356
Growth rate (cm/years)	7.2 ± 1.5	5.1 ± 0.4	0.001
Calcium (mg/dL)	9.9 ± 0.3	10.0 ± 0.3	0.19**2**
Phosphorus (mg/dL)	4.8 ± 0.5	4.7 ± 0.5	0.231
PTH (pg/mL)	44.8 ± 16.3	31.0 ± 11.9	0.001
25(OH)D (ng/mL)	25.4 ± 8.6	28.2 ± 7.4	0.187

*Student’s *t*-test

**Table 2:** Biochemical characteristic according to the season of the year in CPP and control groups (*M* ± SDS)

CPP group (*n* = 78)	Winter (*n* = 20)	Spring (*n* = 18)	Summer (*n* = 22)	Autumn (*n* = 18)	*p* value*
Calcium (mg/dL)	9.9 ± 0.3	9.9 ± 0.3	10.0 ± 0.4	9.9 ± 0.2	0.785
Phosphorus (mg/dL)	4.7 ± 0.4	4.9 ± 0.3	4.7 ± 0.4	4.8 ± 0.5	0.284
25 (OH)D (ng/mL)	23.1 ± 7.0	23.7±7.8	32.4 ± 8.4	23.0 ± 5.9	0.034
PTH (pg/mL)	48.8 ± 15.2^#^	47.3 ± 15.5^#^	37.7 ± 14.9^#^	50.2 ± 17.1^#^	0.009

Control group (*n* = 127)	Winter (*n* = 38)	Spring (*n* = 25)	Summer (*n* = 26)	Autumn (*n* = 38)	*p* value*
Calcium (mg/dL)	10.1 ± 0.3	10.0 ± 0.2	10.0 ± 0.2	9.9 ± 0.4	0.358
Phosphorus (mg/dL)	4.6 ± 0.5	4.6 ± 0.3	4.6 ± 0.5	4.7 ± 0.5	0.608
25 (OH)D (ng/mL)	26.2 ± 6.7	26.0 ± 6.4	36.3 ± 7.7	26.5 ± 7.1	0.001
PTH (pg/mL)	29.4 ± 12.6^#^	30.5 ± 9.3^#^	23.6 ± 6.5^#^	35.1 ± 12.9^#^	0.003

*Kruskal Wallis test

^#^(*p* < 0.05) between groups (Mann-Whitney’s *U*-test)

**Table 3:** Biochemical determinations according to vitamin D status in PPC and control groups (*M* ± SDS)

CPP group (*n* = 78)	Deficiency (*n* = 12)	Insufficiency (*n* = 39)	Sufficiency (*n* = 27)	*p* values*
Calcium (mg/dL)	9.8 ± 0.3	9.9 ± 0.3	10.0 ± 0.3	0.232
Phosphorus(mg/dL)	4.9 ± 0.5	4.7 ± 0.4	4.7 ± 0.5	0.169
25 (OH)D (ng/mL)	14.9 ± 2.8	23.5 ± 3.0	36.7 ± 5.1	0.001
PTH (pg/mL)	59.5 ± 15.4^#^	47.1 ± 17.5^#^	36.2 ± 14.6^#^	0.001

Control group (*n* = 137)	Deficiency (*n* = 16)	Insufficiency (*n* = 63)	Sufficiency (*n* = 58)	*p* values*
Calcium (mg/dL)	9.9 ± 0.4	10.1 ± 0.2	10.1 ± 0.3	0.726
Phosphorus (mg/dL)	4.8 ± 0.6	4.6 ± 0.6	4.6 ± 0.6	0.291
25 (OH)D (ng/mL)	15.8 ± 2.8	24.9 ± 2.6	35.1 ± 4.6	0.001
PTH (pg/mL)	33.9 ± 13.3^#^	33.8 ± 14.6^#^	27.3 ± 11.6^#^	0.001

*Kruskal–Wallis test

^#^(*p* < 0.05) between groups (Mann–Whitney’s *U*-test)

**Table 4:** Clinical data and concentrations of gonadotropin according to vitamin D status in PPC group (*M* ± SDS)

	Deficiency (*n* = 12)	Insufficiency (*n* = 39)	Sufficiency (*n* = 27)	*p* values*
Age (years)	7.42 ± 0.60	7.40 ± 0.60	7.58 ±0.43	0.571
BMI SDS	0.30 ± 0.67	0.29 ± 0.71	0.38 ± 0.68	0.665
Growth rate (cm/years)	7.16 ± 1.45	7.35 ± 1,31	7.19 ± 1.13	0.846
Bone age (years)	8.96 ± 0.87	9.04 ± 0.90	9.10 ± 0.80	0.722
BA-CA (years)	1.44 ± 0.75	1.64 ± 0.63	1.52 ± 0.70	0.668
Basal estradiol	26.33 ± 9.77	26.63 ± 8.44	26.40 ± 11.51	0.998
Basal LH (UI/L)	0.67 ± 0.38	0.56 ± 0.51	0.81 ± 0.62	0.760
LH peak (UI/L)	14.01 ± 6.55	13.22 ± 9.62	14.09 ± 7.67	0.962
Basal FSH (UI/L)	3.59 ± 0.94	3.06 ± 1.37	3.09 ± 1.67	0.698
FSH peak (UI/L)	19.31 ± 7.06	16.19 ± 4.26	18.76 ± 7.16	0.222

*Kruskal–Wallis test

## Data Availability

The datasets generated during and/or analysed during the current study are available from the corresponding author on reasonable request.

## References

[1] Hossein-Nezhad A, Holick MF (2013). Vitamin D for health: a global perspective. Mayo Clin Proc.

[2] Palermo NE, Holick MF (2014). Vitamin D, bone health, and other health benefits in pediatric patients. J Pediatr Rehabil Med.

[3] Holick MF (2007). Vitamin D deficiency. N Engl J Med.

[4] Vierucci F, Del Pistoia M, Fanos M, Gori M, Carlone G, Erba P (2013). Vitamin D status and predictors of hypovitaminosis D in Italian children and adolescents: a cross-sectional study. Eur J Pediatr.

[5] Durá-Travé T, Gallinas-Victoriano F, Chueca-Guindulain MJ, Berrade-Zubiri S (2015). Vitamin D deficiency among children and adolescents with normal nutricional status. Nutr Hosp.

[6] Houghton LA, Gray AR, Harper MJ, Winichagoon P, Pongcharoen T, Gowachirapant S (2014). Vitamin D status among Thai school children and the association with 1,25-dihydroxyvitamin D and parathyroid hormone levels. PLoS ONE.

[7] Chung IH, Kim HJ, Chung S, Yoo S (2014). Vitamin D deficiency in Korean children: prevalence, risk factors, and the relationship with parathyroid hormone levels. Ann Pediatr Endocrinol Metab.

[8] Durá-Travé T, Gallinas-Victoriano F (2016). Seasonal variations in calcidiol and parathyroid hormone levels in healthy children and adolescents in Navarre, Spain: a cross-sectional study. JRSM Open.

[9] Stagi S, Cavalli L, Ricci S, Mola M, Cinzia M, Seminara S (2015). Parathyroid hormone levels in healthy children and adolescents. Horm Res Paediatr.

[10] Lerchbaum E, Obermayer-Pietsch B (2012). Vitamin D and fertility: a systematic review. Eur J Endocrinol.

[11] Shahrokhi SZ, Ghaffari F, Faranak KF (2016). Role of vitamin D in female reproduction. Clin Chim Acta.

[12] Lee HS, Kim YJ, Shim YS, Jeong HR, Kwon E, Hwang JS (2014). Associations between serum vitamin D levels and precocious puberty in girls. Ann Pediatr Endocrinol Metab.

[13] Zhao Y, Long W, Du C, Yang H, Wu S, Ning Q (2018). Prevalence of vitamin D deficiency in girls with idiopathic central precocious puberty. Front Med.

[14] Villamor E, Marin C, Mora-Plazas M, Baylin A (2011). Vitamin D deficiency and age at menarche: a prospective study. Am J Clin Nutr.

[15] Duhil de Bénazé G, Brauner R, Souberbielle JC (2017). There is no association between vitamin D status and characteristics of central precocious puberty in girls. Eur J Pediatr.

[16] Durá-Travé T, Gallinas-Victoriano F, Malumbres-Chacon M, Ahmed-Mohamed L, Chueca-Guindulain MJC, Berrade-Zubiri S (2021). Clinical data and basal gonadotropins in the diagnosis of central precocious puberty in girls. Endocr Connect.

[17] Durá-Travé T, Gallinas-Victoriano F, Urretavizcaya-Martinez M, Ahmed-Mohamed L, Guindulain MJC, Berrade-Zubiri S (2019). Assessment of body composition changes during a combined intervention for the treatment of childhood obesity. Nutrition.

[18] Tanner JM, Whitehouse RH, Marshall WA, Healy MJR, Goldstein H (1983). Assessment of skeletal maturity and prediction of adult height (TW2 method).

[19] Ferrández A, Baguer L, Labarta JI, Labena C, Mayayo E, Puga B et al (2005) Longitudinal pubertal growth according to age at pubertal study of normal Spanish children from birth to adulthood. Pediatric Endocrinology. Zaragoza, Spain: Fundación Andrea Prader. https://www.gastroinf.es/nutritional/. Accessed Jan 2021

[20] Holick MF, Binkley NC, Bischo-Ferrari HA, Gordon CM, Hanley DA, Heaney RP (2011). Endocrine Society. Evaluation, treatment, and prevention of vitamin D deficiency: an Endocrine Society clinical practice guideline. J Clin Endocrinol Metab.

[21] Holick MF, Binkley NC, Bischo-Ferrari HA, Gordon CM, Hanley DA, Heaney RP (2012). Guidelines for preventing and treating vitamin D deficiency and insuficiency revisited. J Clin Endocrinol Metab.

[22] Allgrove J, Brook C, Clayton PO, Brown R (2005). The parathyroid and disorders of calcium metabolism. Clinical pediatric endocrinology.

[23] Rovner AJ, O’Brien KO (2005). Hypovitaminosis D among healthy children in the United States: a review of the current evidence. Arch Pediatr Adolesc Med.

[24] Karaguzel G, Dilber B, Can G, Okten A, Deger O, Holick MF (2014). Seasonal vitamin D status of healthy schoolchildren and predictors of low vitamin D status. J Pediatr Gastroenterol Nutr.

[25] Pacífico L, Anania C, Osborn JF, Ferraro F, Bonci E, Olivero E (2011). Low 25(OH)D3 levels are associated with total adiposity, metabolic syndrome, and hypertension in Caucasian children and adolescents. Eur J Endocrinol.

[26] Turer CB, Lin H, Flores G (2013). Prevalence of vitamin D deficiency among overweight and obese US children. Pediatrics.

[27] Radhakishun N, van Vliet M, von Rosenstiel I, Weijer O, Diamant M, Beijnen J (2015). High prevalence of vitamin D insufficiency/deficiency in Dutch multi-ethnic obese children. Eur J Pediatr.

[28] Durá-Travé T, Gallinas-Victoriano F, Chueca-Guindulain MJ, Berrade-Zubiri S (2017). Prevalence of hypovitaminosis D and associated factors in obese Spanish children. Nutr Diabetes.

[29] Hansen L, Tjonneland A, Koster B, Brot C, Andersen R, Cohen AS (2018). Vitamin D status and seasonal variation among Danish children and adults: A descriptive study. Nutrients.

[30] Vigano P, Lattuada D, Mangioni S, Ermellino L, Vignali M, Caporizzo E (2006). Cycling and early pregnant endometrium as a site of regulated expression of the vitamin D system. J Mol Endocrinol.

[31] Kinuta K, Tanaka H, Moriwake T, Aya K, Kato S, Seino Y (2000). Vitamin D is an important factor in estrogen biosynthesis of both female and male gonads. Endocrinology.

[32] Bacopoulou F, Kolias E, Efthymiou V, Antonopoulos CN, Charmandari E (2017). Vitamin D predictors in polycystic ovary syndrome: a meta-analysis. Eur J Clin Invest.

[33] Fang F, Ni K, Cai Y, Shang J, Zhang X, Xiong C (2017). Effect of vitamin D supplementation on polycystic ovary syndrome: a systematic review and meta-analysis of randomized controlled trials. Complement Ther Clin Pract.

[34] Dicken CL, Israel DD, Davis JB, Sun Y, Shu J, Hardin J (2012). Peripubertal vitamin D(3) deficiency delays puberty and disrupts the estrous cycle in adult female mice. Biol Reprod.

[35] Bachrach LK (2001). Acquisition of optimal bone mass in childhood and adolescence. Trends Endocrinol Metab.

